# IL-17A Promotes Intracellular Growth of *Mycobacterium* by Inhibiting Apoptosis of Infected Macrophages

**DOI:** 10.3389/fimmu.2015.00498

**Published:** 2015-09-30

**Authors:** Andrea Cruz, Paula Ludovico, Egidio Torrado, José Bernardo Gama, Jeremy Sousa, Joana Gaifem, Rui Appelberg, Fernando Rodrigues, Andrea M. Cooper, Jorge Pedrosa, Margarida Saraiva, António G. Castro

**Affiliations:** ^1^Life and Health Sciences Research Institute (ICVS), School of Health Sciences, University of Minho, Braga, Portugal; ^2^ICVS/3B’s – PT Government Associate Laboratory, University of Minho, Braga, Portugal; ^3^Trudeau Institute, Saranac Lake, NY, USA; ^4^Department of Immunophysiology, University of Porto, Porto, Portugal

**Keywords:** IL-17, p53, apoptosis, mycobacteria, macrophages

## Abstract

The fate of infected macrophages is a critical aspect of immunity to mycobacteria. By depriving the pathogen of its intracellular niche, apoptotic death of the infected macrophage has been shown to be an important mechanism to control bacterial growth. Here, we show that IL-17 inhibits apoptosis of *Mycobacterium bovis* BCG- or *Mycobacterium tuberculosis*-infected macrophages thus hampering their ability to control bacterial growth. Mechanistically, we show that IL-17 inhibits p53, and impacts on the intrinsic apoptotic pathway, by increasing the Bcl2 and decreasing Bax expression, decreasing cytochrome *c* release from the mitochondria, and inhibiting caspase-3 activation. The same effect of IL-17 was observed in infected macrophages upon blockade of p53 nuclear translocation. These results reveal a previously unappreciated role for the IL-17/p53 axis in the regulation of mycobacteria-induced apoptosis and can have important implications in a broad spectrum of diseases where apoptosis of the infected cell is an important host defense mechanism.

## Introduction

*Mycobacterium tuberculosis* is an intracellular pathogen responsible for the death of ~1.5 million people every year (1). Macrophages are the first cells to be infected by *M. tuberculosis* and provide the bases for a sequence of microbicidal actions which contain the infection (2). A growing body of evidence points to the ability to manipulate apoptosis, a form of programed cell death, as an important determinant of mycobacterial pathogenesis, although conflicting results support either inhibition or activation of apoptosis as a virulence mechanism (3, 4). Indeed, several studies suggest that the induction of apoptosis is unique to avirulent strains of mycobacteria, acting as a mechanism to prevent the spread of infection, by allowing antigen cross-presentation in favor of the host (5–8). In contrast, other authors argue that only virulent strains of *M. tuberculosis* induce apoptosis of infected cells (3, 9, 10). These apparent discrepancies may be due to experimental conditions, with *in vivo* data suggesting that both apoptosis and necrosis of infected cells occur, depending on the anatomical location and timing of infection (3).

Among the factors dictating the fate of mycobacterially infected cells is the cytokine composition of the extracellular milieu. Specifically, tumor necrosis factor (TNF)-α induces while interleukin (IL)-10 inhibits apoptosis of the mycobacterially infected macrophages (11, 12). Although *M. tuberculosis*-infected macrophages are exposed to many other cytokines produced during the immune response, the impact of these cytokines on apoptosis of infected cells remains unclear. In this context, IL-17 is interesting, as this cytokine produced by T helper (Th)17 cells during chronic viral infection leads to the upregulation of the pro-survival proteins Bcl-xl and Bcl2 resulting in reduced apoptosis of infected cells; this, in turn, contributes to viral persistence and pathogenesis (13). IL-17 was also shown to promote survival of airway macrophages upon allergen-induced airway inflammation (14) and of fibroblast-like synoviocytes during chronic inflammation, through signal-transducer and activator of transcription protein (STAT)-3 activation (15). Moreover, IL-17 and Th17 cells have been linked to cancer, although both pro- and anti-tumorigenic activities have been ascribed to this cytokine (16–18).

The evidence supporting a role for IL-17 in modulating the apoptotic pathway, together with the early production of IL-17 following *M. tuberculosis* infection (19), prompted us to investigate the role of IL-17 in the modulation of apoptosis in mycobacterially infected macrophages. Using mouse primary bone marrow-derived macrophages (BMDM) infected with *M. tuberculosis* or *M. bovis* BCG, we show that in the presence of IL-17 macrophages are more permissive to *Mycobacterium* growth consistent with a reduced apoptotic death of the infected macrophages. Mechanistically, we show that IL-17 interferes with the master regulator of apoptosis p53, alters the transcriptional profile of Bax and Bcl2 and inhibits cytochrome *c* release.

## Materials and Methods

### Animals

Eight- to 12-week-old female C57BL/6 mice obtained from Charles River (Barcelona, Spain) were used. All experimental protocols involving mice were performed according to the European Union Directive 86/609/EEC, and previously approved by the national authority *Direção Geral de Alimentação e Veterinária*.

### Reagents and bacteria

Recombinant mouse IL-17A (R&D Systems) and the specific p53 chemical inhibitor cyclic pifithrin-alpha (PFT-α; Sigma) were used at 100 ng/mL and 30 μM, respectively, based on a dose response curve (data not shown). *M. bovis* BCG Pasteur or *M. tuberculosis* H37Rv (a kind gift of Pere-Juan Cardona, Barcelona) stocks were prepared as previously described (20).

### Culture of mouse BMDM

Bone marrow-derived macrophages were generated and infected at a multiplicity of infection (MOI) of two bacteria per macrophage as described (20, 21). Cells were treated as described in the figure legends. To determine the number of viable bacteria, macrophage cultures were lysed with 0.1% saponin and the bacterial suspensions were serially diluted and plated onto 7H11 agar medium. Bacterial colony formation was counted after 3 weeks of incubation at 37°C. To determine the viability of BMDM in culture, the medium was aspirated and the adherent cells incubated for 10 min with 2.5 mg/mL of protease type XXV (Sigma) to digest any dead cells. Next, cetrimide (Sigma) was added to lyse the viable cells and a suspension of the cell nuclei was obtained and counted using a Neubauer chamber.

### Nitrites quantification

Nitrite production by macrophages was determined by the Griess assay (22).

### Cytokine analysis by ELISA

Tumor necrosis factor and IL-10 were measured in the culture supernatants using commercial kits (eBioscience) according to the manufacturer’s specifications.

### Immunofluorescence

Bone marrow-derived macrophages were cultured in cover-slips placed at the bottom of the 24-well incubation plates, infected, and treated according to the description in the figure legends. At specific time points after infection, cells were fixed in 2% buffered paraformaldehyde (PFA) and probed with rabbit anti-human/mouse specific for active caspase 3 (R&D Systems) or anti-mouse specific for p53 (Cell Signaling), followed by Alexa Fluor 594-conjugated goat anti-rabbit or anti-mouse IgG (Molecular Probes), respectively. 4′,6-Diamino-2-phenylindole hydrochloride (DAPI) (Molecular Probes) was used to detect nuclei. Quantification of mean intensity of p53 protein was performed using the regions of interest (ROI) Manager application of the ImageJ program, which quantifies the mean intensity of selected areas. A total of five different fields for each cover-slip was analyzed. The mean intensity was calculated by selecting the ROI using the freehand selection tool. The number of caspase-3 positive cells was determined by acquiring five different fields of the cover-slips and counting 300 cells for each condition. The percentage of caspase-3 positive cells was calculated by dividing the number of caspase-3 positive cells by the total number of cells.

### Quantitative real-time PCR analysis

Total RNA from cultured BMDM was extracted and reverse-transcribed as described previously (20). Mouse p53, Bcl2, and Bax gene mRNA expression was quantified using specific primer probes (ABI) (Mm01731287_m1; Mm00477631_m1 and Mm00432051_m1, respectively) and normalized to the hypoxanthine guanine phosphoribosyl transferase (HPRT) mRNA levels (Mm00446968_m1).

### Cytochrome *c* release

The protocol was performed as previously described (23). Briefly, cells were permeabilized with digitonin buffer (50 μg/mL of digitonin in PBS with 100 mM KCl), fixed in 2% (w/v) buffered-PFA in PBS (pH 7.4), for 30 min at room temperature, rinsed, resuspended in PBS, and stored at 4°C. When all time points had been collected, 10^6^ cells were incubated in blocking buffer for 1 h, at room temperature, stained with mouse purified anti-cytochrome *c* antibody (BD Pharmingen) overnight at 4°C, followed by the secondary anti-mouse IgG1-PE antibody (BioLegend) for 20 min. Stained cells were run in a LSRII flow cytometer and analyzed with FlowJo software (Tristar).

### Western blot

Protein extracts were prepared, resolved in 12% SDS-PAGE, and western blot performed as described previously (24). Primary antibodies were actin (JLA20, developed by Lin JJC and obtained from the Developmental Studies Hybridoma Bank, developed under the auspices of the National Institute of Child Health and Human Development and maintained by The University of Iowa, Department of Biology), Bax, Bcl2, and caspase-3 (all Cell Signaling). Secondary antibodies were from Southern Biotech or Santa Cruz. The same membrane was used to detect Bax and Bcl2. Total and cleaved caspase-3 was detected in the same membrane. Chemiluminescence detection system SuperSignal West Femto (Thermo Scientific) and the Universal Hood II (Bio-Rad) were used as a detection system. Band intensity was quantified using QuantityOne (Bio-Rad).

### Statistical analysis

The results are given as mean ± SE. Statistical significance was calculated by using Student’s *t* test, or one-way ANOVA with Tukey’s post-test. Values of *p* ≤ 0.05 were considered significant.

## Results

### IL-17 renders macrophages more permissive to *mycobacterium* growth

**Figure 1:**
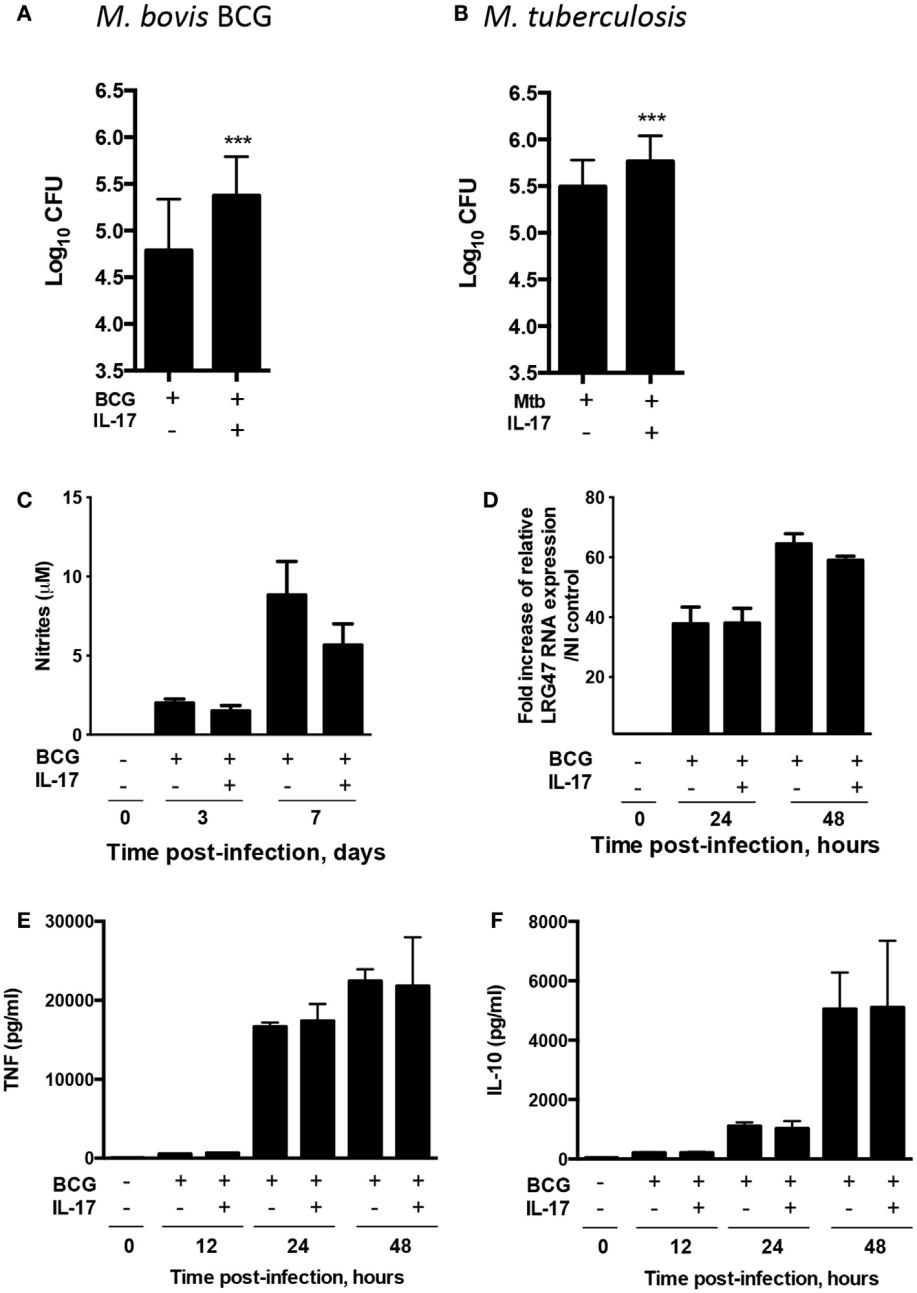
**IL-17 promotes growth of *M. bovis* BCG and *M. tuberculosis* in infected BMDM**. BMDM were infected with *M. bovis* BCG (A) or with *M. tuberculosis* **(B)** and treated (+) or not (−) with IL-17. **(A)** Four days post-infection, the bacterial load was assessed. Fifty out of 50 independent experiments are represented in the graph. The mean-fold increase in CFUs induced by IL-17 is 0.4 ± 0.2 log. **(B)** Three days post-infection, the bacterial load was assessed. Eleven out of 11 independent experiments are represented in the graph. The mean-fold increase in CFUs induced by IL-17 is 0.28 ± 0.18 log. Significance determined by Student’s *t* test (***p < 0.001). BMDM were left uninfected or infected with *M. bovis* BCG and treated (+) or not (−) with IL-17. **(C)** Nitrites production by was measured in the supernatants of the cultures by the Griess method. **(D)** The mRNA expression of LRG47 was quantified by real-time PCR using the primers (sense 5′-CTCTGGATCAGGGTTTGAGGAGTA-3′; anti-sense 5′-GGAACT GTGATGGTTTCATGATA-3′) and probes (5′-LCred640-AGGTCCACAGACAGCGTCACTCGG-P-3′; 5′-AACCAGAGAGCCTCACCAGG GAGCTGA-FL-3′) and normalized to HPRT. The fold increase of LRG47 mRNA expression over NI control was calculated. Represented are the mean ± SE of three independent experiments. **(E,F)** At different time points post-infection, supernatants were harvested and the production of TNF **(E)** and IL-10 **(F)** assessed by immunoassay. Data point represents a mean of *n* = 6. Results are from one representative out of three independent experiments.

IL-17 production is induced upon *M. bovis* BCG and *M. tuberculosis* infection, with γδ T cells as the major cellular source during the early stage of the infection (19, 25). However, the impact of this cytokine on mycobacterially infected macrophages remains to be investigated. To address this, mouse primary BMDM were infected with *M. bovis* BCG in the presence or absence of IL-17 and, 4 days after infection, the number of viable bacteria present in each condition was determined. A significantly higher number of viable bacteria were consistently found in macrophages stimulated with IL-17 (Figure 1A). Importantly, this effect was not specific for *M. bovis* BCG as the ability of macrophages to control *M. tuberculosis* was also significantly hampered by IL-17 (Figure 1B). These data suggest that IL-17 renders mycobacterially infected macrophages more permissive to bacterial growth. Notably, IL-17 did not interfere with basic macrophage microbicidal mechanisms, such as nitric oxide production (Figure 1C) or LRG47 expression, a marker for phagolysosome formation (Figure 1D). Since TNF and IL-10 play important roles on the regulation of apoptosis in mycobacterially infected macrophages (11, 12), a possible regulation of these molecules by IL-17 was next investigated. However, IL-17 did not impact the production of TNF (Figure 1E) or of IL-10 (Figure 1F) observed in macrophages upon *M. bovis* BCG infection.

### IL-17 interferes with p53 in mycobacterially infected macrophages

**Figure 2:**
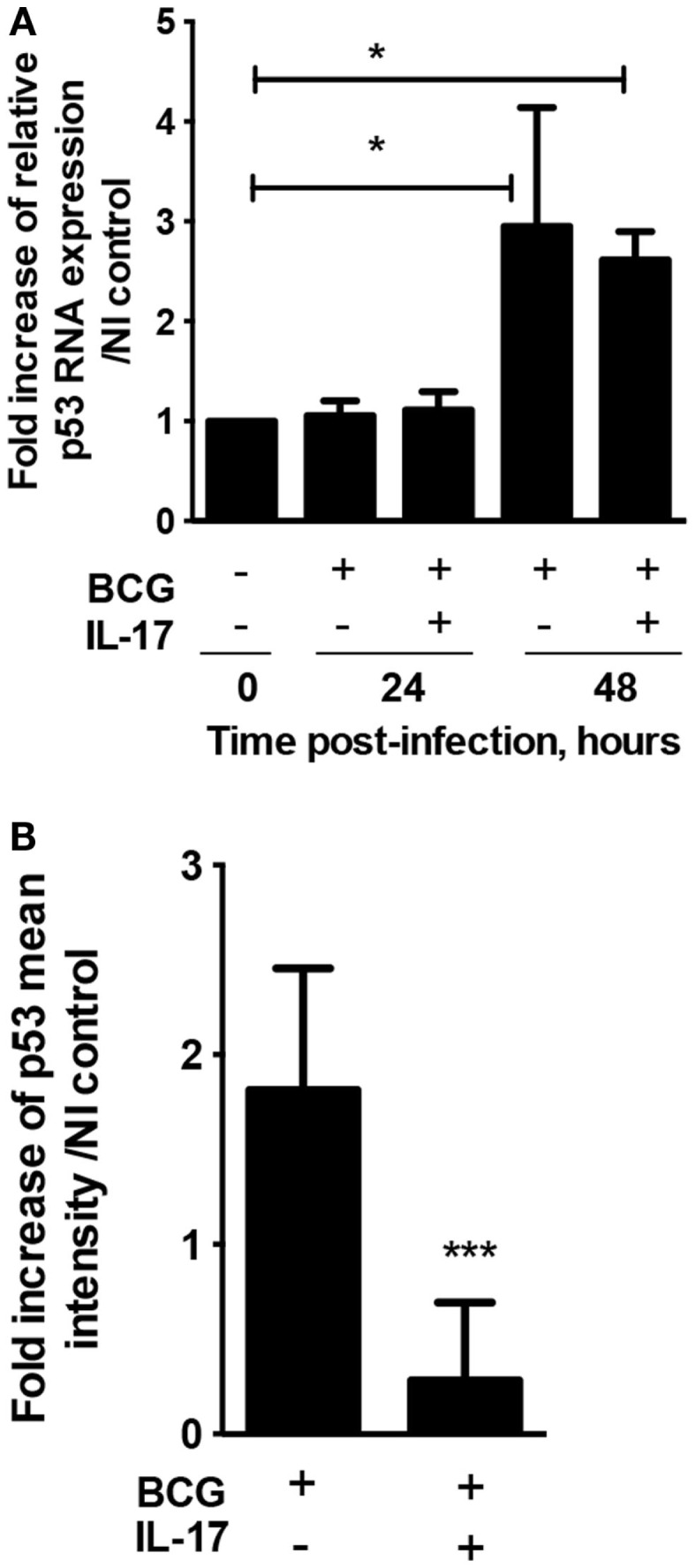
**IL-17 inhibits the upregulation of p53 observed during infection of BMDM with *M. bovis* BCG**. BMDM were infected with *M. bovis* BCG and treated (+) or not (−) with IL-17. **(A)** At the indicated time points, the expression of p53 was assessed by quantitative real-time PCR and normalized against HPRT. The fold increase of p53 mRNA expression over non-infected (NI) control was calculated. **(B)** Two days post-infection p53 levels were assessed by immunofluorescence and the fold increase of p53 mean intensity of cells over NI control calculated using ROI Manager application of the ImageJ program. The CFU controls for the represented experiments are plotted in Figure 1A. Representative images used for the calculations are in Figure S1 in Supplementary Material. Represented are the mean ± SE of three independent experiments each of them performed for triplicate conditions. Significance determined by Student’s *t* test (***p < 0.001).

Activation of the apoptotic pathway is thought to be a host defensive mechanism against mycobacterial pathogens (26). IL-17 has been shown to modulate apoptosis in viral infection (13) and in cancer (17, 18), but its role in the context of mycobacterial infection inducing apoptosis has not been addressed yet. Since IL-17 has been shown to down-regulate the activity of the apoptosis master regulator p53 (27, 28), we investigated whether IL-17 is able to modulate p53 in mycobacterially infected macrophages. While an upregulation of p53 transcription upon infection with *M. bovis* BCG was observed 48 h post-infection, this was not affected by the presence of IL-17 (Figure 2A). In contrast, the increase in p53 protein levels as measured by immunofluorescence, observed as early as 2 days post-*M. bovis* BCG infection, was abrogated by IL-17 (Figure 2B; Figure S1 in Supplementary Material). These data show that IL-17 down-regulates p53 at the protein level, suggesting that it may interfere with apoptosis of infected cells. Further, this interference may be the pathway by which IL-17 impacts the bacterial growth control.

### IL-17 inhibits apoptosis in mycobacterially infected macrophages

**Figure 3:**
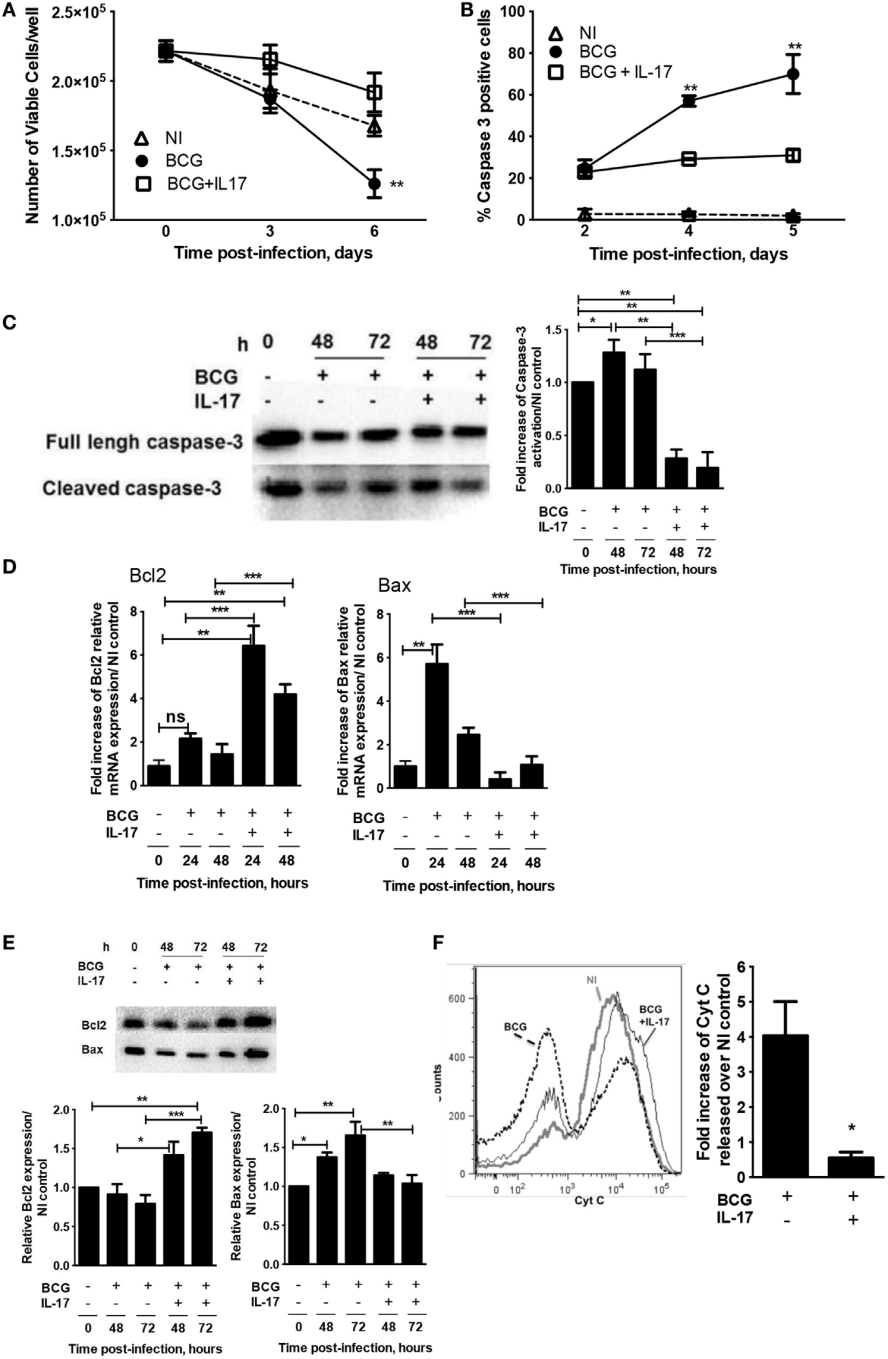
**IL-17 inhibits macrophage apoptosis induced by mycobacterial infection**. BMDM were infected with *M. bovis* BCG in the presence or absence of IL-17 as indicated. **(A)** On days 2 and 7 post-infection, the cell viability was assessed by enumerating nuclei. **(B)** On days 2, 4, and 5 post-*M. bovis* BCG infection, activation of caspase-3 was assessed by immunofluorescence. **(C)** At 48 or 72 h post-*M. bovis* BCG infection, full-length and cleaved forms of caspase-3 protein were determined by western blot. **(D,E)** At 24 or 48 h post-*M. bovis* BCG infection, the Bcl2 and Bax mRNA or at 48 or 72 h post-*M. bovis* BCG infection, the protein were determined by real-time PCR or western blot respectively.**(F)** Cytochrome *c* release from the mitochondria was determined 2 days post-infection by flow cytometry. The fold increase of cytochrome *c* released over NI control was calculated for each independent experiment. Representative images used for **(A)** and **(B)** are in Figure S2 in Supplementary Material. Represented are the mean ± SE of four independent experiments. Significance determined by one-way ANOVA **(A,B)** or Student’s *t* test **(D–F)** (*p < 0.05; **p < 0.01; ***p < 0.001).

Given that IL-17 modulated p53 protein, this cytokine could impact on the induction of apoptotic programed cell death, an important antimicrobial mechanism operating upon mycobacterial infection (26). Thus, the role of IL-17 on the survival of infected macrophages was investigated. First, the viability of BMDM at different times post-infection with *M. bovis* BCG was measured. As expected, the survival of infected macrophages decreased over time as compared to non-infected (NI) cells. However, the presence of IL-17 increased the survival of infected macrophages to levels similar to those of NI cells (Figure 3A; Figure S2A in Supplementary Material). Next, BMDM were infected with *M. bovis* BCG in the absence or presence of IL-17 and the activation of caspase-3, a major apoptotic effector caspase, was determined by immunofluorescence microscopy. We found that the presence of IL-17 resulted in a reduction in the percentage of caspase-3-positive macrophages through day 5 of infection with *M. bovis* BCG (Figure 3B; Figure S2B in Supplementary Material) or with *M. tuberculosis* (Figure S2C in Supplementary Material). Additionally, and in line with the immunofluorescence data, we observed a decrease in the protein levels of activated (cleaved) caspase-3 upon addition of IL-17 to BCG-infected macrophages, as determined by western blot (Figure 3C). Since our previous data (Figure 2) showed that IL-17 reduced p53 protein and since p53 is a master regulator of pro- and anti-apoptotic molecules (29), namely Bax and Bcl2, we next explored the impact of IL-17 on these molecules. Upon infection with *M. bovis* BCG, the transcription of Bcl2 was not much affected when compared to NI cells, whereas that of Bax was significantly increased (Figure 3D). In the presence of IL-17, a marked increase of Bcl2 transcription accompanied by a decrease of Bax mRNA was observed (Figure 3D). The increased Bcl2 transcription observed in the presence of IL-17 was confirmed at the protein level, as measured by western blot (Figure 3E). In contrast, addition of IL-17 did not affect the amount of Bax protein (Figure 3E). In all, in the presence of IL-17, the ratio of Bcl2/Bax proteins was significantly increased, suggesting an anti-apoptotic activity for IL-17. The ratio of Bcl2 to Bax is an important component of the intrinsic apoptotic pathway that regulates the permeabilization of the outer mitochondrial membrane, and thereby the release of cytochrome *c*. Therefore, the release of cytochrome *c* in *M. bovis* BCG-infected macrophages in the presence or absence of IL-17 was determined. We found that cytochrome *c* release was observed upon infection of macrophages, in line with the activation of the intrinsic apoptotic pathway (Figure 3F). Further, it was clear that cytochrome *c* release induced by BCG was abrogated in the presence of IL-17 (Figure 3F). These data suggest that IL-17 inhibits the intrinsic apoptotic pathway by altering the Bcl2/Bax ratio and the release of cytochrome *c*.

### Modulation of p53 activity impacts mycobacterial growth in macrophages

**Figure 4:**
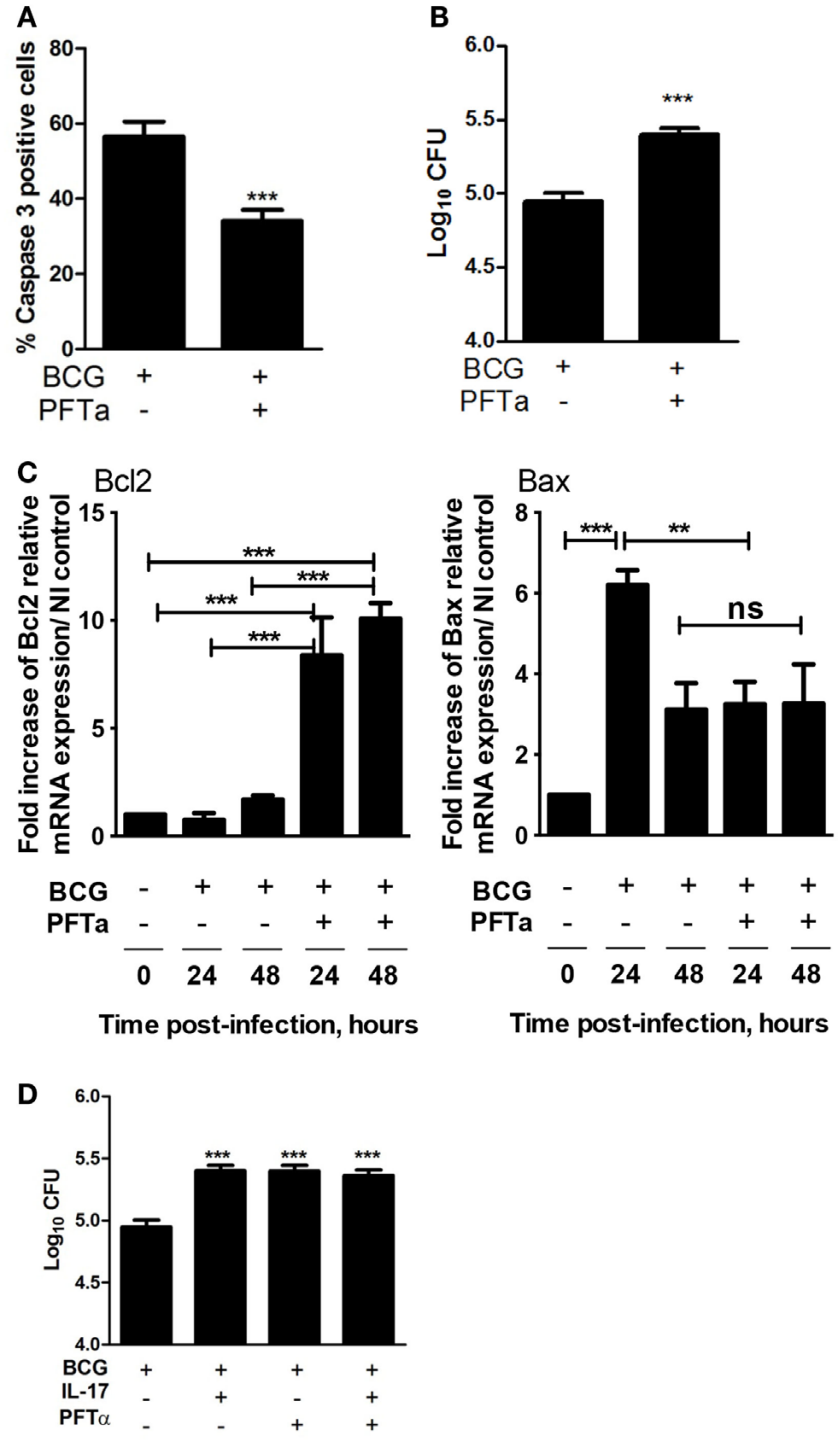
**Blockade of p53 impairs apoptosis of infected macrophages and bacterial growth control**. BMDM were infected with *M. bovis* BCG in the presence (+) or absence (−) of the p53 inhibitor PFTα. Four days post-infection **(A)** caspase-3 activation was assessed by immunofluorescence and **(B)** the bacterial load was assessed as indicated before. Representative images used for the calculations are in Figure S3 in Supplementary Material. **(C)** At 24 or 48 h post-*M. bovis* BCG infection, the Bcl2 and Bax mRNA was determined by real-time PCR. The fold increase of Bcl2 or Bax mRNA over the NI control was calculated for each independent experiment. **(D)** BMDM were left uninfected or infected with *M. bovis* BCG in the presence or absence of IL-17 or of PFTα as indicated. Four days post-infection the bacterial load was assessed as indicated before. Represented are the mean ± SE of three independent experiments. Significance determined by Student’s *t* test (***p < 0.001).

As IL-17 decreases the capacity of macrophages to limit bacterial growth (Figure 1), reduces availability of p53, and limits apoptosis in mycobacterially infected macrophages, we hypothesized that IL-17 impacts mycobacterial growth via modulation of p53 activity. To test this hypothesis, we chemically inhibited the nuclear translocation of p53 during infection of macrophages with *M. bovis* BCG. Blocking the nuclear activity of p53 led to a significant decrease on the percentage of caspase 3-positive cells (Figure 4A and Figure S3A in Supplementary Material) accompanied by a significant increase in the number of bacteria (Figure 4B). Also, we observed that PFT-α addition to *M. bovis* BCG-infected macrophages increased the expression of the Bcl2, whereas that of Bax was not significantly altered (Figure 4C). The effect of PFT-α in increasing the number of bacteria was not further potentiated by IL-17 (Figure 4D), suggesting that p53 is a downstream mediator of IL-17 in this experimental setting. All our data show that IL-17, by modulating cell survival, impacts mycobacterial control by macrophages.

## Discussion

The importance of apoptosis in infection and of its modulation by intracellular pathogens is established. Apoptosis of mycobacterially infected cells is an important microbicidal mechanism, preventing the spread of the pathogen and promoting antigen cross-presentation (26). Whether the virulence of the infecting mycobacterial strain plays a role in promoting or evading apoptosis of the host cells is still a matter of debate (3, 4). However, it is clear that understanding the mechanisms underlying the fate of *M. tuberculosis*-infected macrophages is of critical importance, as therein may lay the potential to intervene. The programed cell death pathways in mycobacterially infected cells are regulated by host-encoded molecules, including eicosanoids (5, 6) and cytokines (11, 12). Our study adds a new layer to the complexity of this regulation, by reporting a pathway wherein IL-17 functions as an anti-apoptotic mediator in mycobacterially infected cells through its action on p53 and inhibition of the mitochondrial apoptotic pathway.

We dissected the early events of the apoptotic pathway initiated by mycobacterial infection. Following *M. bovis* BCG infection, p53 transcription occurred and there was an increase in the percentage of p53-positive cells, in agreement with previous studies (11). This was accompanied by a reduction in the Bcl2/Bax ratio, the activation of caspase-3 and an increase in cytochrome *c* release. All these pro-apoptotic events were down-regulated by IL-17 activity, placing this cytokine as a novel apoptotic modulator in the context of mycobacterial infection. Several lines of evidence support a link between IL-17 and apoptosis, for example, in the modulation of Fas-Fas ligand induced apoptosis in the context of a viral infection (13), of fibroblast-like synoviocytes apoptosis, through the activation of STAT-3 (15), and in inhibition of p53 transcription in a lymphoma cell line (27). As we now show, in the context of mycobacteria infection, IL-17 acts by decreasing the levels of p53 protein in *M. bovis* BCG-infected BMDM. This modulation is likely taking place at the post-transcriptional level since alterations of p53 transcription were not observed in our experimental setting. An important control mechanism for p53 regulation is through ubiquitination, with several E3 ligases described for p53 (30). Act1, the key transducer of IL-17 receptor signaling, is also an E3 ubiquitin ligase, which shares conserved residues with those known to ubiquitinate p53 and activate its proteasomal degradation (31). It is, therefore, tempting to speculate that IL-17 may be promoting the degradation of p53 through proteasome degradation. It will be important in future to further dissect the IL-17-driven upstream events leading to the observed alterations, particularly those happening immediately after IL-17R triggering. Mechanistically, it is possible that the effect of IL-17 in blocking apoptosis of the infected cell requires other molecules produced during infection. Although the balance between TNF and IL-10 within mycobacterially infected macrophages is important in the occurrence of apoptosis of the infected cell (12), we did not observe an impact of IL-17 in the amount of secreted TNF or IL-10 produced.

Elucidating the mechanisms by which IL-17-mediates p53 modulation is central not only in the context of intracellular infections, but also considering the fact that several studies involving the axis IL-17/p53 in cancer have been undertaken (27, 28, 32) and that IL-17-based therapies are being developed (33).

## Conflict of Interest Statement

The authors declare that the research was conducted in the absence of any commercial or financial relationships that could be construed as a potential conflict of interest.

## Supplementary Material

The Supplementary Material for this article can be found online at http://journal.frontiersin.org/article/10.3389/fimmu.2015.00498
