# Supplementary material for: IL-17A Promotes Intracellular Growth of Mycobacterium by Inhibiting Apoptosis of Infected Macrophages
Source: Front Immunol. 2015 Sep 30;6:498. doi: 10.3389/fimmu.2015.00498 (PMC4588696; doi:10.3389/fimmu.2015.00498)
Supplement: Supplementary file 1 [file Image_1.PDF]

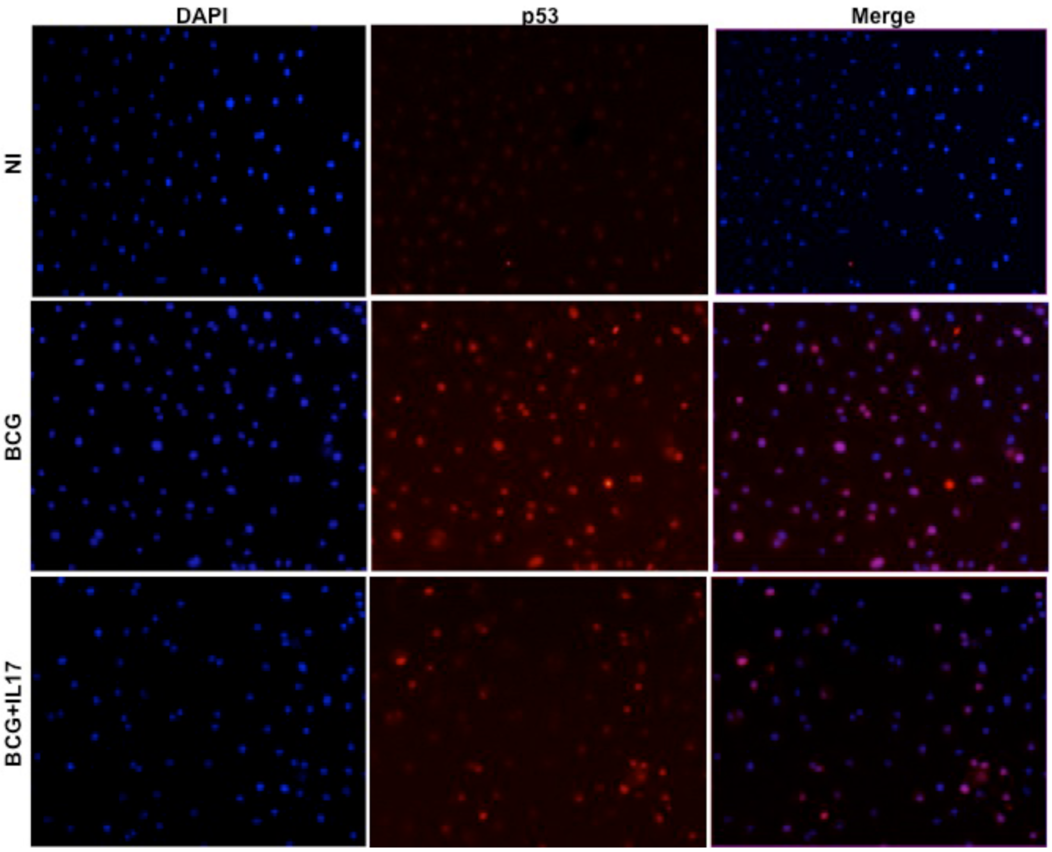

Figure S1 | Representative image of the immunofluorescence used to calculate p53+ cells plotted in Figure 2b.
