# Supplementary material for: IL-17A Promotes Intracellular Growth of Mycobacterium by Inhibiting Apoptosis of Infected Macrophages
Source: Front Immunol. 2015 Sep 30;6:498. doi: 10.3389/fimmu.2015.00498 (PMC4588696; doi:10.3389/fimmu.2015.00498)
Supplement: Supplementary file 2 [file Image_2.PDF]

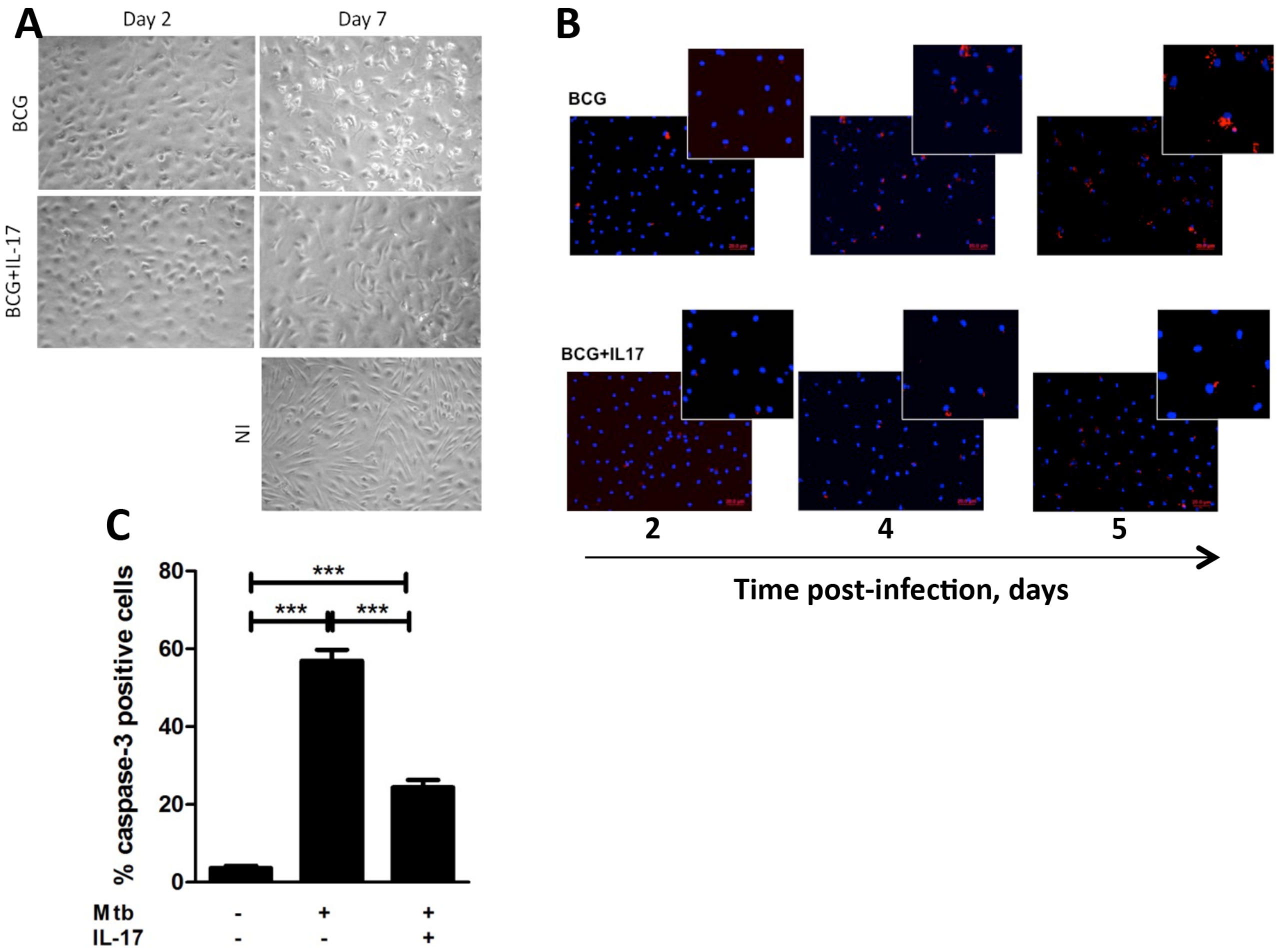

Figure S2 | BMDM were left uninfected or infected with M. bovis BCG (**A**,**B**) or M. tuberculosis (**C**) in the presence or absence of IL-17 as indicated. (**A**) Representative photographs of the cultures were taken on day 2 or 7 as indicated. (**B**) Representative images of the immunofluorescence used to calculate caspase3+ cells plotted in Figure 3B. (**C**) IL-17 decreases caspase3 activation upon M. tuberculosis infection. On day 3 post-M. tuberculosis infection in the presence (+) or absence (-) of IL-17, activation of caspase-3 was assessed by immunofluorescence. Represented are the mean±SD of 3 independent experiments. The CFU controls for the represented experiments are plotted in Figure 1a. Significance determined by one-way ANOVA (***, $p<0.001$).
