# Supplementary material for: IL-17A Promotes Intracellular Growth of Mycobacterium by Inhibiting Apoptosis of Infected Macrophages
Source: Front Immunol. 2015 Sep 30;6:498. doi: 10.3389/fimmu.2015.00498 (PMC4588696; doi:10.3389/fimmu.2015.00498)
Supplement: Supplementary file 3 [file Image_3.PDF]

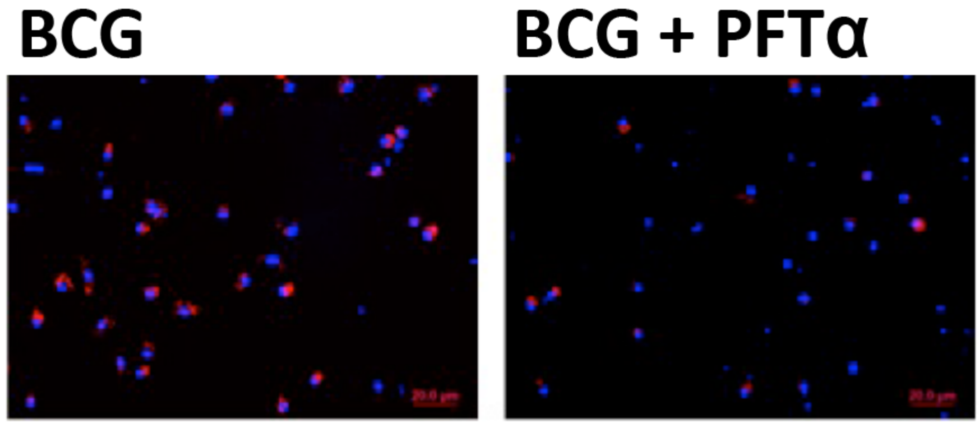

Figure S3 | BMDM were left uninfected or infected with M. bovis BCG in the presence or absence of IL-17 or of PFTα as indicated. Representative images of the immunofluorescence used to calculate caspase-3 positive cells plotted in Figure 4A.
